# Statistical analysis of real-time PCR data

**DOI:** 10.1186/1471-2105-7-85

**Published:** 2006-02-22

**Authors:** Joshua S Yuan, Ann Reed, Feng Chen, C Neal Stewart

**Affiliations:** 1 Department of Plant Sciences, University of Tennessee, Knoxville, TN 37996, USA; 2 University of Tennessee Institute of Agriculture Genomics Hub, University of Tennessee, Knoxville, TN 37996, USA; 3 Statistical Consulting Center, University of Tennessee, Knoxville, TN 37996, USA

## Abstract

**Background:**

Even though real-time PCR has been broadly applied in biomedical sciences, data processing procedures for the analysis of quantitative real-time PCR are still lacking; specifically in the realm of appropriate statistical treatment. Confidence interval and statistical significance considerations are not explicit in many of the current data analysis approaches. Based on the standard curve method and other useful data analysis methods, we present and compare four statistical approaches and models for the analysis of real-time PCR data.

**Results:**

In the first approach, a multiple regression analysis model was developed to derive ΔΔCt from estimation of interaction of gene and treatment effects. In the second approach, an ANCOVA (analysis of covariance) model was proposed, and the ΔΔCt can be derived from analysis of effects of variables. The other two models involve calculation ΔCt followed by a two group *t-*test and non-parametric analogous Wilcoxon test. SAS programs were developed for all four models and data output for analysis of a sample set are presented. In addition, a data quality control model was developed and implemented using SAS.

**Conclusion:**

Practical statistical solutions with SAS programs were developed for real-time PCR data and a sample dataset was analyzed with the SAS programs. The analysis using the various models and programs yielded similar results. Data quality control and analysis procedures presented here provide statistical elements for the estimation of the relative expression of genes using real-time PCR.

## Background

**Figure 1:**
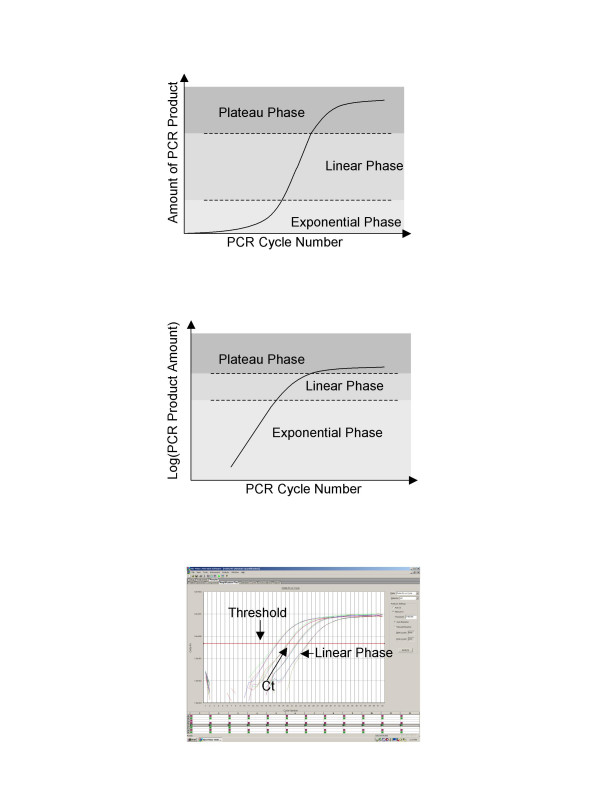
**Real-time PCR**. (A) Theoretical plot of PCR cycle number against PCR product amount is depicted. Three phases can be observed for PCRs: exponential phase, linear phase and plateau phase. (B) shows a theoretical plot of PCR cycle number against logarithm PCR product amount. Panel (C) is the output of a serial dilution experiment from an ABI 7000 real-time PCR instrument.

Real-time PCR is one of the most sensitive and reliably quantitative methods for gene expression analysis. It has been broadly applied to microarray verification, pathogen quantification, cancer quantification, transgenic copy number determination and drug therapy studies [1-4]. A PCR has three phases, exponential phase, linear phase and plateau phase as shown in Figure 1. The exponential phase is the earliest segment in the PCR, in which product increases exponentially since the reagents are not limited. The linear phase is characterized by a linear increase in product as PCR reagents become limited. The PCR will eventually reach the plateau phase during later cycles and the amount of product will not change because some reagents become depleted. Real-time PCR exploits the fact that the quantity of PCR products in exponential phase is in proportion to the quantity of initial template under ideal conditions [5,6]. During the exponential phase PCR product will ideally double during each cycle if efficiency is perfect, i.e. 100%. It is possible to make the PCR amplification efficiency close to 100% in the exponential phases if the PCR conditions, primer characteristics, template purity, and amplicon lengths are optimal.

Both genomic DNA and reverse transcribed cDNA can be used as templates for real-time PCR. The dynamics of PCR are typically observed through DNA binding dyes like SYBR green or DNA hybridization probes such as molecular beacons (Strategene) or Taqman probes (Applied Biosystems) [2]. The basis of real-time PCR is a direct positive association between a dye with the number of amplicons. As shown in Figure (1B and 1C), the plot of logarithm 2-based transformed fluorescence signal versus cycle number will yield a linear range at which logarithm of fluorescence signal correlates with the original template amount. A baseline and a threshold can then be set for further analysis. The cycle number at the threshold level of log-based fluorescence is defined as Ct number, which is the observed value in most real-time PCR experiments, and therefore the primary statistical metric of interest.

Real-time PCR data are quantified absolutely and relatively. Absolute quantification employs an internal or external calibration curve to derive the input template copy number. Absolute quantification is important in case that the exact transcript copy number needs to be determined, however, relative quantification is sufficient for most physiological and pathological studies. Relative quantification relies on the comparison between expression of a target gene versus a reference gene and the expression of same gene in target sample versus reference samples [7].

Since relative quantification is the goal for most for real-time PCR experiments, several data analysis procedures have been developed. Two mathematical models are very widely applied: the efficiency calibrated model [7,8] and the ΔΔCt model [9]. The experimental systems for both models are similar. The experiment will involve a control sample and a treatment sample. For each sample, a target gene and a reference gene for internal control are included for PCR amplification from serially diluted aliquots. Typically several replicates are used for each diluted concentration to derive amplification efficiency. PCR amplification efficiency can be either defined as percentage (from 0 to 1) or as time of PCR product increase per cycle (from 1 to 2). Unless specified as percentage amplification efficiency (PE), we refer the amplification efficiency (E) to PCR product increase (1 to 2) in this article. The efficiency-calibrated model is a more generalized ΔΔCt model. Ct number is first plotted against cDNA input (or logarithm cDNA input), and the slope of the plot is calculated to determine the amplification efficiency (E). ΔCt for each gene (target or reference) is then calculated by subtracting the Ct number of target sample from that of control sample. As shown in Equation 1, the ratio of target gene expression in treatment versus control can be derived from the ratio between target gene efficiency (E_target_) to the power of target ΔCt (ΔCt_target_) and reference gene efficiency (E_reference_) to the power of reference ΔCt (ΔCt_reference_). The ΔΔCt model can be derived from the efficiency-calibrated model, if both target and reference genes reach their highest PCR amplification efficiency. In this circumstance, both target efficiency (E_target_) and control efficiency (E_control_) equals 2, indicating amplicon doubling during each cycle, then there would be the same expression ratio derived from 2^-ΔΔCt ^[7,9].

Ratio=(Etarg⁡et)ΔCttarg⁡et(Ereference)ΔCtreference     Equation 1
 MathType@MTEF@5@5@+=feaafiart1ev1aaatCvAUfKttLearuWrP9MDH5MBPbIqV92AaeXatLxBI9gBaebbnrfifHhDYfgasaacH8akY=wiFfYdH8Gipec8Eeeu0xXdbba9frFj0=OqFfea0dXdd9vqai=hGuQ8kuc9pgc9s8qqaq=dirpe0xb9q8qiLsFr0=vr0=vr0dc8meaabaqaciaacaGaaeqabaqabeGadaaakeaacqWGsbGucqWGHbqycqWG0baDcqWGPbqAcqWGVbWBcqGH9aqpdaWcaaqaaiabcIcaOiabdweafnaaBaaaleaacqWG0baDcyGGHbqycqGGYbGCcqGGNbWzcqWGLbqzcqWG0baDaeqaaOGaeiykaKYaaWbaaSqabeaacqqHuoarcqWGdbWqcqWG0baDdaWgaaadbaGaemiDaqNagiyyaeMaeiOCaiNaei4zaCMaemyzauMaemiDaqhabeaaaaaakeaacqGGOaakcqWGfbqrdaWgaaWcbaGaemOCaiNaemyzauMaemOzayMaemyzauMaemOCaiNaemyzauMaemOBa4Maem4yamMaemyzaugabeaakiabcMcaPmaaCaaaleqabaGaeuiLdqKaem4qamKaemiDaq3aaSbaaWqaaiabdkhaYjabdwgaLjabdAgaMjabdwgaLjabdkhaYjabdwgaLjabd6gaUjabdogaJjabdwgaLbqabaaaaaaakiaaxMaacaWLjaGaemyrauKaemyCaeNaemyDauNaemyyaeMaemiDaqNaemyAaKMaem4Ba8MaemOBa4MaeeiiaaccbiGae8xmaedaaa@79AC@

*Whereas *Δ*Ct*_*t*arg*et *_= *Ct*_*control *_- *Ct*_*treatment *_and Δ*Ct*_*reference *_= *Ct*_*control *_- *Ct*_*treatment*_

*Ratio *= 2^-ΔΔ*Ct *^    *Equation 2*

*Whereas *ΔΔ*Ct *= Δ*Ct*_*reference *_- Δ*Ct*_*t*arg*et*_

Even though both the efficiency-calibrated and ΔΔCt models are widely applied in gene expression studies, not many papers have thorough discussions of the statistical considerations in the analysis of the effect of each experimental factor as well as significance testing. One of the few studies that employed substantial statistical analysis used the REST^® ^program [8]. The software presented in this article is based on the efficiency-calibrated model and employed randomization tests to obtain the significance level. However, the article did not provide a detailed model for the effects of different experimental factors involved. Another statistical study of real-time PCR data used a simple linear regression model to estimate the ratio through Ct calculation [10]. However, the logarithm-based fluorescence was used as the dependent variable in the model, which we believe does not adequately reflect the nature of real-time PCR data. It follows that Ct should be the dependent variable for statistical analysis, because it is the outcome value directly influenced by treatment, concentration and sample effects. Both studies used the efficiency-calibrated models. Despite the publication of these two methods, many research articles published with real-time PCR data actually do not present *P *values and confidence intervals [11-13]. We believe that these statistics are desirable to facilitate robust interpretation of the data.

*A priori*, we consider the confidence interval and *P *value of ΔΔCt data to be very important because these directly influence the interpretation of ratio. Without a proper statistical modeling and analysis, the interpretation of real-time PCR data may lead the researcher to false positive conclusions, which is especially potentially troublesome in clinical applications. We hereby developed four statistical methodologies for processing real-time PCR data using a modified ΔΔCt method. The statistical methodologies can be adapted to other mathematical models with modifications. SAS programs implementing the methodologies and data control are presented with real-time PCR practitioners in mind for turnkey data analysis. Standard deviations, confidence levels and *P *values are presented directly from the SAS output. We also included analysis of the sample data set and SAS programs for the analysis in the online supplementary materials.

## Results and discussion

### Data quality control

From the two mathematical models for relative quantification of real-time PCR data, we observe disparities between data quality standards. For efficiency-calibrated method, the author who described this procedure [7] assumed that the amplification efficiency for each gene (target and reference) is the same among different experimental samples (treatment and control). In contrast, whereas an amplification efficiency of 2 is not required, the ΔΔCt method is more stringent by assuming that all reactions should reach an amplification efficiency of 2. In other words, the amount of product should double during each cycle [9]. Moreover, the ΔΔCt method assumes that the PCR amplification efficiency for each sample will be 2, if PCRs for one set of the samples reaches full amplification efficiency. However, this assumption neglects the effect of different cDNA samples.

Data quality could be examined through a correlation model. Even though examining the correlation between Ct number and concentration can provide an effective quality control, a better approach might be to examine the correlation between Ct and the logarithm (base 2) transformed concentration of template, which should yield a significant simple linear relationship for each gene and sample combination. For example, for a target gene in the control sample, the Ct number should correlate with the logarithm transformed concentration following the simple linear regression model in equation 3. In the equation, *X*_*lcon *_represents the logarithm transformed concentration, *β*_0 _represents the intercept of the regression line, and *β*_*con *_represents the slope of the regression line [14]. The acceptable real-time PCR data should have two features from the regression analysis. First, the slope should not be significantly different from -1. Second, the slopes for all four combinations of genes and samples as shown in Table 1 should not be significantly different from one another. A SAS program was developed to perform the data quality control in Program1_QC.sas (additional file 1).

**Table 1:** The sample real-time PCR data for analysis. In this data set, there two types of samples (treatment and control); two genes (reference and target); and four concentrations of each combination of gene and sample. For data quality control and ANCOVA analysis, the real-time PCR sample data set can be grouped in four groups according to the combination of sample and gene. The Control-Target combination effect was named group 1, Treatment-Target group 2, Control-Reference group 3 and Treatment-Reference group 4.

**Replicate**	**Sample**	**Gene**	**Concentration**	**Ct**	**Group (Class)**
1	Control	Target	10	23.1102	1
2	Control	Target	10	22.9003	1
3	Control	Target	10	22.8972	1
1	Control	Target	2	26.5801	1
2	Control	Target	2	26.2139	1
3	Control	Target	2	26.0606	1
1	Control	Target	0.4	28.1125	1
2	Control	Target	0.4	28.1899	1
3	Control	Target	0.4	27.5949	1
1	Control	Target	0.08	30.2772	1
2	Control	Target	0.08	30.4667	1
3	Control	Target	0.08	30.7571	1
1	Treatment	Target	10	21.7813	2
2	Treatment	Target	10	21.7564	2
3	Treatment	Target	10	21.641	2
1	Treatment	Target	2	23.7965	2
2	Treatment	Target	2	23.7571	2
3	Treatment	Target	2	23.724	2
1	Treatment	Target	0.4	26.3794	2
2	Treatment	Target	0.4	26.2542	2
3	Treatment	Target	0.4	25.9621	2
1	Treatment	Target	0.08	28.5479	2
2	Treatment	Target	0.08	28.3894	2
3	Treatment	Target	0.08	28.3416	2
1	Control	Reference	10	19.7415	3
2	Control	Reference	10	19.494	3
3	Control	Reference	10	19.3906	3
1	Control	Reference	2	21.9838	3
2	Control	Reference	2	22.4435	3
3	Control	Reference	2	22.57	3
1	Control	Reference	0.4	24.8109	3
2	Control	Reference	0.4	24.4327	3
3	Control	Reference	0.4	24.2342	3
1	Control	Reference	0.08	26.7319	3
2	Control	Reference	0.08	26.8206	3
3	Control	Reference	0.08	26.822	3
1	Treatment	Reference	10	18.4468	4
2	Treatment	Reference	10	18.8227	4
3	Treatment	Reference	10	18.3061	4
1	Treatment	Reference	2	21.2568	4
2	Treatment	Reference	2	21.0956	4
3	Treatment	Reference	2	20.8473	4
1	Treatment	Reference	0.4	23.2322	4
2	Treatment	Reference	0.4	22.9577	4
3	Treatment	Reference	0.4	23.2415	4
1	Treatment	Reference	0.08	25.4817	4
2	Treatment	Reference	0.08	25.608	4
3	Treatment	Reference	0.08	25.5675	4

*Ct = β*_0 _+ *β*_*con*_*X*_*lcon *_+ *ε *    *Equation 3*

**Figure 2:**
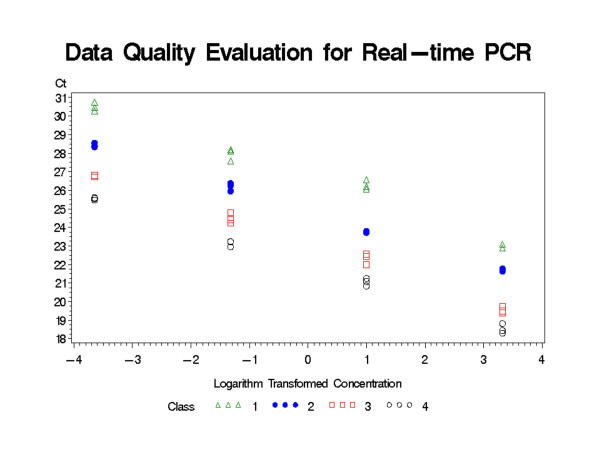
**Data quality control**. The four classes represent four different combinations of sample and gene, which are reference gene in control sample, target gene in control sample, reference gene in treatment sample, and target gene in treatment sample. Each class should derive a linear correlation between Ct and logarithm transformed concentration pf PCR product with a slope of -1.

The input data is grouped as shown in Table 1 and additional file 2. Each combination of gene and sample was classified in one group named from 1 to 4. The SAS procedure Proc Mixed was used for performing simple linear regression for each group based on the model described above. The 95% confidence levels for slopes were estimated, which are expected not be significantly different from -1. The abbreviated SAS output for the analysis of a sample data set is presented in SASOutput.doc (additional file 3). Slopes for Ct and logarithm transformed concentrations for all four groups were not significantly different from -1 based on 95% confidence level. In addition to the numeric output, the program also provides a visualization of data quality as shown in Figure 2, where the Ct number is plotted against logarithm transformed template concentration. A simple linear relationship should be observed between the Ct number and logarithm transformed concentration.

### Multiple regression model

Several effects need to be taken in to consideration in the ΔΔCt method, namely, the effect of treatment, gene, concentration, and replicates. If we consider these effects as quantitative variables and have the Ct number relating to these multiple effects and their interactions, we can develop a multiple regression model as follows in Equation 4.

*Ct *= *β*_0 _+ *β*_*con*_*X*_*icon *_+ *β*_*treat*_*X*_*itreat *_+ *β*_*gene*_*X*_*igene *_+ *β*_*contreat*_*X*_*icon*_*X*_*itreat *_+ *β*_*congene*_*X*_*icon*_*X*_*igene *_+ *β*_*genetreat*_*X*_*igene*_*X*_*itreat *_+ *β*_*congenetreat*_*X*_*icon*_*X*_*itreat*_*X*_*igene *_+ *ε *    *Equation 4*

In this model, Ct is the true dependent, the *β*_0 _is the intercept, *β*_*x*_s are the regression coefficients for the corresponding X (independent) terms, and *ε *is the error term [14]. The model considers the effect of concentration, treatment, gene and their interactions. We are principally interested in the interaction between gene and treatment, which addresses the degree of the Ct differences between target gene and reference gene in treated vs. control samples: i.e., ΔΔCt. ΔΔCt can therefore be estimated from the different combinations values of β_genetreat_. The four groups in Table 1 also represent the options of combinational effects of treatment and gene. The goal is to statistically test for differences between target and reference genes in treatment vs. control samples. Therefore, the null hypothesis is the Ct differences between target and reference genes will be the same in treatment vs control samples, which can be represented by combinational effect (CE) as: CE1-CE3 = CE2-CE4. An alternative formula will be: CE1-CE2-CE3+CE4 = 0, which will yield an estimation of ΔΔCt. If the null hypothesis is not rejected, then the ΔΔCt would not be significantly different from 0, otherwise, the ΔΔCt can be derived from the estimation of the test. In this way, we can perform a test of different combinational effects of β_genetreat _and estimate the ΔΔCt from it. As shown in the ΔΔCt formula in Equation 2, if a ΔΔCt is equal to 0, the ratio will be 1, which indicates no change in gene expression between control and treatment.

### A SAS program for multiple regression model

SAS procedure PROC GLM was used for ΔΔCt estimation in Program2_MR.sas in additional file 4. The multiple regression model is stated in a model statement. The combinational effect of gene and treatment are evaluated in the estimate and contrast statement. The null hypothesis of CE1-CE2-CE3+CE4 = 0 is tested in the contrast statement and the parameter estimation yield the ΔΔCt value. The SAS input file is available in additional file 5 and the SAS output for the multiple regression is in SASOutput.doc (additional file 3).

The SAS output gives a very comprehensive analysis of the data. We are interested in two aspects of the analysis. First, we want to test whether the ΔΔCt value is significantly different from 0 at *P *= 0.05. If the ΔΔCt is not significantly different from 0, then we conclude the treatment does not have a significant effect on target gene expression; otherwise, the inverse is concluded. If the effect is significant, we are interested in the standard deviation of ΔΔCt value, from which we can derive the ratio of gene expression as discussed later. The SAS output provides the point estimation (-0.6848) and standard error (-0.1185) for the ΔΔCt. PROC GLM or PROC MIXED are interchangeable in this application. If the experiments involve multiple biological replicates, replicate effect can also be considered through modifying the SAS program. Then the estimation will be the combined effect of gene, treatment and replicate.

### Analysis of covariance and SAS code

Another way to approach the real-time PCR data analysis is by using an analysis of covariance (ANCOVA). A simplified model can be derived from transforming the data into a grouped data as shown in Table 1 and additional file 2 resulting in Equation 5.

*Ct *= *β*_0 _+ *β*_*con*_*X*_*icon *_+ *β*_*group*_*X*_*igroup *_+ *β*_*groupcon*_*X*_*igroup*_*X*_*icon *_+ *ε*.     *Equation 5*

We are interested in two questions here. First, are the covariance adjusted averages among the four groups equal? Second, what is the Ct difference of target gene value between treatment and control sample after corrected by reference gene? In this case, the null hypothesis will be (μ2-μ1)-(μ4-μ3) = 0, and the test will yield a parameter estimation of ΔΔCt as shown in the Program3_ANCOVA.sas (additional file 6).

The SAS code implementing the ANCOVA model is similar to that of multiple regression model. Either SAS procedures PROC GLM or PROC MIXED can be employed to implement the ANCOVA model; and we used PROC MIXED here. The class statement defines which variables will be grouped for significance testing. In this case, the variables are concentration and group, and ANCOVA assumes that these are co-varying in nature. The contrast and estimate statements were used to contrast the group effect, which will yield ΔΔCt (-0.6848), as well as its standard error (0.1185) and 95% confidence interval (-0.9262, -0.4435). The SAS output with both confidence level and *P *value is presented in SASOutputs.doc (additional file 3).

### Simplified alternatives – *T*-test and wilcoxon two group test

More simplified alternatives can be used to analyze real-time data with biological replicates for each experiment. The primary assumption with this approach is that the additive effect of concentration, gene, and replicate can be adjusted by subtracting Ct number of target gene from that of reference gene, which will provide ΔCt as shown in Table 2. The ΔCt for treatment and control can therefore be subject to simple *t*-test, which will yield the estimation of ΔΔCt.

**Table 2:** ΔCt calculation. The table presents the calculation of ΔCt, which is derived from subtracting Ct number of reference gene from that of the target gene. Con stands for concentration.

**Sample**	**Gene**	**Con**	**Ct**	**Sample**	**Gene**	**Con**	**Ct**	**ΔCt**
Control	Target	10	23.1102	Control	Reference	10	19.7415	3.3687
Control	Target	10	22.9003	Control	Reference	10	19.494	3.4063
Control	Target	10	22.8972	Control	Reference	10	19.3906	3.5066
Control	Target	2	26.5801	Control	Reference	2	21.9838	4.5963
Control	Target	2	26.2139	Control	Reference	2	22.4435	3.7704
Control	Target	2	26.0606	Control	Reference	2	22.57	3.4906
Control	Target	0.4	28.1125	Control	Reference	0.4	24.8109	3.3016
Control	Target	0.4	28.1899	Control	Reference	0.4	24.4327	3.7572
Control	Target	0.4	27.5949	Control	Reference	0.4	24.2342	3.3607
Control	Target	0.08	30.2772	Control	Reference	0.08	26.7319	3.5453
Control	Target	0.08	30.4667	Control	Reference	0.08	26.8206	3.6461
Control	Target	0.08	30.7571	Control	Reference	0.08	26.822	3.9351
Treatment	Target	10	21.7813	Treatment	Reference	10	18.4468	3.3345
Treatment	Target	10	21.7564	Treatment	Reference	10	18.8227	2.9337
Treatment	Target	10	21.641	Treatment	Reference	10	18.3061	3.3349
Treatment	Target	2	23.7965	Treatment	Reference	2	21.2568	2.5397
Treatment	Target	2	23.7571	Treatment	Reference	2	21.0956	2.6615
Treatment	Target	2	23.724	Treatment	Reference	2	20.8473	2.8767
Treatment	Target	0.4	26.3794	Treatment	Reference	0.4	23.2322	3.1472
Treatment	Target	0.4	26.2542	Treatment	Reference	0.4	22.9577	3.2965
Treatment	Target	0.4	25.9621	Treatment	Reference	0.4	23.2415	2.7206
Treatment	Target	0.08	28.5479	Treatment	Reference	0.08	25.4817	3.0662
Treatment	Target	0.08	28.3894	Treatment	Reference	0.08	25.608	2.7814
Treatment	Target	0.08	28.3416	Treatment	Reference	0.08	25.5675	2.7741

As a non-parametric alternative to the *t*-test, a Wilcoxon two group test can also be used to analyze the two pools of ΔCt values. Two of the assumptions for *t-*test are that the both groups of ΔCt will have Gaussian distributions and they will have equal variances. However, these assumptions are not valid in many real-time PCR experiments using realistically small sample sizes. Therefore a distribution-free Wilcoxon test will be a more robust and appropriate alternative in this case [15].

A SAS program has been developed for both *t-*test and Wilcoxon two group test as shown in the attached program Program4_TW.sas (additional file 7). The SAS procedures TTEST and UNIVARIATE were used to analyze the data. The SAS Macro 'moses.sas' [15] in additional file 8 has been employed to derive the confidence levels. The SAS input file is in additional file 9 and the SAS output for sample data analysis is available in SASOutput.doc (additional file 3). Since the estimate of difference derives from subtracting treatment from control sample, the actual ΔΔCt should be the inverse of the output estimate.

### Comparison of four approaches and data presentation

A comparison of the four approaches is presented in Table 3. Multiple regression and ANCOVA yield exactly the same result for ΔΔCt estimation, because both methods employ the same mathematical approach for parameter estimation. The *t*-test provides the same point estimation of ΔΔCt, however, the standard error is slightly greater, which leads to a larger confidence interval. Wilcoxon two group test provides a slightly smaller estimation of ΔΔCt. The highly similar results from the four approaches validated the models and SAS programs presented. The choice of the models and programs will depend on the experimental design and the stringency and quality of the experiment. However, the most conservative test, owing to its nonparametric nature, is the Wilcoxon two group test, which is distribution-independent.

**Table 3:** The comparison of four approaches. The table listed ΔΔCt, standard error, *P*-value and confidence interval derived from the four methods presented in the article. Neither SAS package nor the macro used provides the standard error for Wilcoxon two group test. We consider confidence interval to be sufficient for further data transformation.

**Model**	**ΔΔCt**	**Standard Error**	***P*-Value**	**Confidence Interval**
Multiple Regression	-0.6848	0.1185	< 0.0001	(-0.4435, -0.9262)
ANCOVA	-0.6848	0.1185	< 0.0001	(-0.4435, -0.9262)
t-test	-0.6848	0.1303	< 0.0001	(-0.4147, -0.955)
Wilcoxon Test	-0.6354		< 0.0001	(-0.4227, -0.8805)

### Data quality control

Many of the current real-time PCR experiments do not include a standard curve design, nor do they use a method to estimate the amplification efficiency. We argue here that real-time PCR data without proper quality controls are not reliable, since the efficiency of real-time PCR could have significant impact on the ratio estimation and dynamic range. For example, if a PCR has a percentage amplification efficiency (PE) of 0.8 (i.e. PCR product will increase 2^0.8 ^times instead of two times per cycle), a ΔCt value of 3 can only be transformed into 5.27 times differences in ratio instead of 8 times. This problem gets amplified when the ΔΔCt or ΔCt values are larger and the amplification efficiency is lower, which could lead to severely skewed interpretations.

We therefore propose two standards for real-time PCR data quality control according to the model using the SAS programs presented in this paper. First, experiments with a serial dilution of template need to be included in order to estimate the amplification efficiency of each gene with each sample. Some researchers assume that the amplification efficiency for each gene is the same in different samples because the same primer pair and amplification conditions are used. However, we found that sample effect does have an impact on the amplification efficiency. In other words, the amplification efficiency could be different for the same gene when amplified from different cDNA template samples. We therefore consider the experimental design with standard curve for each gene and sample combination as the optimal. Second, under optimal conditions, if a plot of the Ct number against the logarithm (2-based) template amount should yield a slope not significantly different from -1, which indicates a nearly 2 amplification efficiency. Even though both efficiency-calibrated model and modified ΔΔCt model tolerates the amplification efficiency lower than 2, it is most reliable to have all the reaction with amplification efficiency approximating 2 through optimizing primer choices, amplicon lengths and experimental conditions. From our experience, maintaining all the amplification efficiency near 2 is the best way to reach equal amplification efficiency among the samples and thus to ensure high quality data. It is also observed that a near 2 amplification efficiency can help to expand the dynamic range of ratio estimation.

### *P*-value, confidence intervals and data presentation

The *P*-value is an important parameter for significance level, and confidence intervals help to establish the reliable range for ΔΔCt estimation. Most of current real-time PCR publications do not present *P*-values and confidence intervals [11-13]. We believe disclosing *P*-values is important when the researchers claim differential expression between the samples or treatments exists. In the program we present, all the *P*-values are derived from testing the null hypothesis that ΔΔCt are equal to 0. Therefore, a small *P*-value indicates that the ΔΔCt is significantly different from 0, which demonstrates a significant effect. The interpretation of a *P-*value will depend on the experimental objectives. For example, at *P *= 0.05 in a treatment versus control experiment, we can claim that the treatment has a significant effect; and in a tissue comparison experiment, we can claim that the gene expression is significantly different among the tissues.

Some publications present a standard deviation of the ratio as a meaningful metric. However, we argue here that the standard deviation of ratio should be derived from the standard deviation of ΔΔCt; and the confidence interval of the ratio should be derived from the confidence interval of ΔΔCt. In other words, the point estimation of ratio should be 2^-ΔΔCt ^and the confidence interval for ratio should be (2^-ΔΔCtHCL^, 2^-ΔΔCtLCL^). Since Ct is the observed value from experimental procedures, it should be the subject of statistical analysis. The practice of performing statistical analysis at ratio directly is not appropriate. The presentation of data needs to refer to the ΔΔCt and subsequently the ratio and confidence intervals derived from 2^-ΔΔCt.^

### Statistical analysis for real-time PCR data with amplification efficiency less than 2

As stated before, the PCR amplification efficiency can be optimized to be approximately 2 with proper amplification primers, RNA quality, and cDNA synthesis protocol. Recent advancements in real-time PCR primer design have allowed easier experimental optimization [16,17]. However, less than ideal real-time PCR data can occur regardless the stringent control of experimental conditions. There are three scenarios for suboptimal real-time PCR data. In the first scenario, all of the PCR reactions have the same amplification efficiency, yet the efficiency differs from 2. In the second scenario, the PCR amplification efficiency differs by gene only. In other words, the amplification efficiency is the same for the same gene in all the biological samples; however, the amplification efficiency varies among the different genes. In the third scenario, the PCR amplification efficiency differs both by gene and by sample. We considered the data in the third scenario as unacceptable as many others have reported [10,18]. In any of these scenarios, the adjusted ΔΔCt can be derived from the ANCOVA model by including the PE in the 'estimate' and 'contrast' statement of the SAS program.

Several approaches have been developed to calculate the amplification efficiency in the low quality data. One of such approach is so called 'dynamic data analysis', in which the fluorescence history of a PCR reaction is employed to calculate the amplification efficiency [19,20]. The advantage of the approach lies in the capacity to analyze low quality data and the economy in cost by avoiding the standard curve. However, due to the mathematical complexity and the reliability controversy, this method is not as widely applied as the traditional standard curve method [10,16,18]. In our method, a standard curve already exists and can be used to derive amplification efficiency (E). Considering the simple linear regression model in Equation 3, if *X*_*lcon *_represents 10 based logarithm transformed concentration, the amplification efficiency (E) is 10^-(1/slope) ^or 10−(1/βcon)
 MathType@MTEF@5@5@+=feaafiart1ev1aaatCvAUfKttLearuWrP9MDH5MBPbIqV92AaeXatLxBI9gBaebbnrfifHhDYfgasaacH8akY=wiFfYdH8Gipec8Eeeu0xXdbba9frFj0=OqFfea0dXdd9vqai=hGuQ8kuc9pgc9s8qqaq=dirpe0xb9q8qiLsFr0=vr0=vr0dc8meaabaqaciaacaGaaeqabaqabeGadaaakeaacqaIXaqmcqaIWaamdaahaaWcbeqaaiabgkHiTiabcIcaOiabigdaXiabc+caVGGaciab=j7aInaaBaaameaacqqGJbWycqqGVbWBcqqGUbGBaeqaaSGaeiykaKcaaaaa@3921@ according to Ramussen 2001 and Pfaffl 2001 [7,21]. In our model, *X*_*lcon *_represents the 2 based logarithm transformed concentration, the amplification efficiency (E) therefore is 2^-(1/slope) ^or 2−(1/βcon)
 MathType@MTEF@5@5@+=feaafiart1ev1aaatCvAUfKttLearuWrP9MDH5MBPbIqV92AaeXatLxBI9gBaebbnrfifHhDYfgasaacH8akY=wiFfYdH8Gipec8Eeeu0xXdbba9frFj0=OqFfea0dXdd9vqai=hGuQ8kuc9pgc9s8qqaq=dirpe0xb9q8qiLsFr0=vr0=vr0dc8meaabaqaciaacaGaaeqabaqabeGadaaakeaacqaIYaGmdaahaaWcbeqaaiabgkHiTiabcIcaOiabigdaXiabc+caVGGaciab=j7aInaaBaaameaacqqGJbWycqqGVbWBcqqGUbGBaeqaaSGaeiykaKcaaaaa@3835@, where the PE can be represented as -(1/β_con_).

In the first scenario discussed above, all PCR amplification have the same efficiency, but the efficiency is not equal to 1. Then the ratio of gene expression can be represented in the following equation.

Ratio=E−ΔΔCt=[2−(1/βcon)]−ΔΔCt=2−ΔΔCt*PE     Equation 6
 MathType@MTEF@5@5@+=feaafiart1ev1aaatCvAUfKttLearuWrP9MDH5MBPbIqV92AaeXatLxBI9gBaebbnrfifHhDYfgasaacH8akY=wiFfYdH8Gipec8Eeeu0xXdbba9frFj0=OqFfea0dXdd9vqai=hGuQ8kuc9pgc9s8qqaq=dirpe0xb9q8qiLsFr0=vr0=vr0dc8meaabaqaciaacaGaaeqabaqabeGadaaakeaacqWGsbGucqWGHbqycqWG0baDcqWGPbqAcqWGVbWBcqGH9aqpcqWGfbqrdaahaaWcbeqaaiabgkHiTiabfs5aejabfs5aejabdoeadjabdsha0baakiabg2da9iabcUfaBjabikdaYmaaCaaaleqabaGaeyOeI0IaeiikaGIaeGymaeJaei4la8ccciGae8NSdi2aaSbaaWqaaGqaciab+ngaJjab+9gaVjab+5gaUbqabaWccqGGPaqkaaGccqGGDbqxdaahaaWcbeqaaiabgkHiTiabfs5aejabfs5aejabdoeadjabdsha0baakiabg2da9iabikdaYmaaCaaaleqabaGaeyOeI0IaeuiLdqKaeuiLdqKaem4qamKaemiDaqNaeiOkaOIaemiuaaLaemyraueaaOGaaCzcaiaaxMaacqWGfbqrcqWGXbqCcqWG1bqDcqWGHbqycqWG0baDcqWGPbqAcqWGVbWBcqWGUbGBcqqGGaaicqGF2aGnaaa@6ABA@

whereas *PE *= -(1/*β*_*con*_), and *ΔΔCt*_*adjust *_= PE*ΔΔCt

In the Equation 6, β_con _is the pooled slope of the plot with Ct against logarithm 2 based concentration. The β_con _can be calculated with a correlation function in SAS as shown in Program5_LowQualityData.sas in Additional file 10. In the second scenario, the amplification efficiency differs by gene only. According to Equation 1, we have the following equation, in which the β_0 _is the pooled slope of the plot of Ct against log_2 _(concentration) for each gene.

Ratio=(Etarg⁡et)ΔCttarg⁡et(Econtrol)ΔCtcontrol=[2−(1/βconTarg⁡et)]ΔCttarg⁡et[2−(1/βconControl)]ΔCtcontrol=2(PEtarg⁡et∗ΔCttarg⁡et−PEcontrol∗ΔCtcontrol)     Equation 7
 MathType@MTEF@5@5@+=feaafiart1ev1aaatCvAUfKttLearuWrP9MDH5MBPbIqV92AaeXatLxBI9gBaebbnrfifHhDYfgasaacH8akY=wiFfYdH8Gipec8Eeeu0xXdbba9frFj0=OqFfea0dXdd9vqai=hGuQ8kuc9pgc9s8qqaq=dirpe0xb9q8qiLsFr0=vr0=vr0dc8meaabaqaciaacaGaaeqabaqabeGadaaakeaacqWGsbGucqWGHbqycqWG0baDcqWGPbqAcqWGVbWBcqGH9aqpdaWcaaqaaiabcIcaOiabdweafnaaBaaaleaacqWG0baDcyGGHbqycqGGYbGCcqGGNbWzcqWGLbqzcqWG0baDaeqaaOGaeiykaKYaaWbaaSqabeaacqqHuoarcqWGdbWqcqWG0baDdaWgaaadbaGaemiDaqNagiyyaeMaeiOCaiNaei4zaCMaemyzauMaemiDaqhabeaaaaaakeaacqGGOaakcqWGfbqrdaWgaaWcbaGaem4yamMaem4Ba8MaemOBa4MaemiDaqNaemOCaiNaem4Ba8MaemiBaWgabeaakiabcMcaPmaaCaaaleqabaGaeuiLdqKaem4qamKaemiDaq3aaSbaaWqaaiabdogaJjabd+gaVjabd6gaUjabdsha0jabdkhaYjabd+gaVjabdYgaSbqabaaaaaaakiabg2da9maalaaabaGaei4waSLaeGOmaiZaaWbaaSqabeaacqGHsislcqGGOaakcqaIXaqmcqGGVaWliiGacqWFYoGydaWgaaadbaGaem4yamMaem4Ba8MaemOBa4MaemivaqLagiyyaeMaeiOCaiNaei4zaCMaemyzauMaemiDaqhabeaaliabcMcaPaaakiabc2faDnaaCaaaleqabaGaeuiLdqKaem4qamKaemiDaq3aaSbaaWqaaiabdsha0jGbcggaHjabckhaYjabcEgaNjabdwgaLjabdsha0bqabaaaaaGcbaGaei4waSLaeGOmaiZaaWbaaSqabeaacqGHsislcqGGOaakcqaIXaqmcqGGVaWlcqWFYoGydaWgaaadbaGaem4yamMaem4Ba8MaemOBa4Maem4qamKaem4Ba8MaemOBa4MaemiDaqNaemOCaiNaem4Ba8MaemiBaWgabeaaliabcMcaPaaakiabc2faDnaaCaaaleqabaGaeuiLdqKaem4qamKaemiDaq3aaSbaaWqaaiabdogaJjabd+gaVjabd6gaUjabdsha0jabdkhaYjabd+gaVjabdYgaSbqabaaaaaaakiabg2da9iabikdaYmaaCaaaleqabaGaeiikaGIaemiuaaLaemyrau0aaSbaaWqaaiabdsha0jGbcggaHjabckhaYjabcEgaNjabdwgaLjabdsha0bqabaWccqGHxiIkcqqHuoarcqWGdbWqcqWG0baDdaWgaaadbaGaemiDaqNagiyyaeMaeiOCaiNaei4zaCMaemyzauMaemiDaqhabeaaliabgkHiTiabdcfaqjabdweafnaaBaaameaacqWGJbWycqWGVbWBcqWGUbGBcqWG0baDcqWGYbGCcqWGVbWBcqWGSbaBaeqaaSGaey4fIOIaeuiLdqKaem4qamKaemiDaq3aaSbaaWqaaiabdogaJjabd+gaVjabd6gaUjabdsha0jabdkhaYjabd+gaVjabdYgaSbqabaWccqGGPaqkaaGccaWLjaGaaCzcaiabdweafjabdghaXjabdwha1jabdggaHjabdsha0jabdMgaPjabd+gaVjabd6gaUjabbccaGGqaciab+Dda3aaa@F60B@

whereas *PE*_*target *_= -(1/*β*_*conTarget*_), *PE*_*control *_= -(1/*β*_*conControl*_), and *ΔΔCt*_*adjust *_= *PE*_target_*ΔCt_*target*_-*PE*_control_*ΔCt_*control*_

In the Equation 7, *β*_*conTarget *_and *β*_*conControl *_are the pooled slope for the plot of Ct against logarithm 2 based concentration for target gene and reference gene respectively. The slopes can be calculated by the Program5_LowQualityData.sas (additional file 10). The *ΔΔCt*_*adjust *_can be calculated with the same program. Theoretically, an equation can also be derived for the third scenario when PCR amplification efficiency differs both by gene and by sample. However, in actual application, we don't consider the data in the third scenario as acceptable due to the significant variation of the amplification efficiency [10,18].

The Program5_LowQuatilityData.sas in additional file 10 provides the solution to derive the adjusted ΔΔCt in the first and second scenarios. A data set with amplification efficiency different by gene is provided in LowQualityData.txt in additional file 11 to illustrate the use of the SAS program. The data set is of lower quality mainly because of the limited number of replicates involved in the experiment. Four steps are involved in calculating the *ΔΔCt*_*adjust*_. The first step is to perform the data quality control test as shown in Methods. From the SAS output, we can conclude that the LowQualityData dataset does not meet the requirements for 2^-ΔΔCt ^method, since one group of PCR has amplification efficiency significantly different from 1 as shown in the data quality control for LowQualityData dataset part of SASOutput.doc (additional file 3).

The second step is to test the equal PCR efficiency (or slope) by observing the Type III sums of squares for lcon and class interaction. A low *p *value will indicate the interaction of different groups of PCR (class) with logarithm transformed concentration, which in turn indicates the unequal slope among different groups of PCR. If all PCR amplification efficiency are equal, then the pooled amplification efficiency can be calculated and integrate into the SAS program for *ΔΔCt*_*adjust *_calculation. In this set of data, the Type III sums of squares has a *p *value smaller than 0.05, and the amplification efficiency are not equal for all PCRs. Tests of equal slopes are then performed for each gene to decide whether PCR amplification efficiency is the same for each gene. For either gene, the amplification efficiency is not significantly different with an α of 0.05. All of the Type III sums of squares outputs can be found in SASOutput.doc (additional file 3).

The next step is to calculate the pooled slope (β_con_) for each gene to derive the percentage amplification efficiency (PE = -(1/β_con_)) for each gene. The pooled slopes are derived based on the correlation between Ct and logarithm 2 based concentrations. The β_con_s for the two genes are -1.0813 and -1.0137 respectively as shown in SASOutput.doc (additional file 3) for the amplification efficiency calculation of LowQualityData dataset. With the β_con_, -(1/β_con_) or PE can be calculated for each gene as 0.925 and 0.987 respectively. The ΔΔCt_adjust _can then be computed with PEs substituting the 1 for each gene in the 'estimate' and 'contrast' statement. The SAS program is as follows in additional file 10.

Title 2 'Calculate the deltadeltaCt with Adjusted efficiency';

**PROC MIXED **data=TR2 Order=Data;

   CLASS Class Con;

   MODEL Ct = Con Class Con*Class/SOLUTION NOINT;

   Contrast 'Intercepts' Class **0.925 **-**0.925 **-**0.987 0.987**;

   Estimate 'Intercepts' Class **0.925 **-**0.925 **-**0.987 0.987**/cl;

**Run**;

The SAS output for the analysis is in SASOutput.doc (additional file 3). The ΔΔCt_adjust _is therefore -1.0901 and the change is significant since *p *value is very small. The ratio can be represented as discussed in the standard ΔΔCt method. The point estimation of the ratio in this example is 2.129, and the 95% confidence interval is (1.926, 2.353).

Overall, in the less optimized PCR reactions, statistical analysis is not only complicated but also compromised for precision and efficiency. Therefore caution should be exercised when performing statistical analysis with the low quality real-time PCR data, which may easily introduce error due to the efficiency adjustment [10,18].

## Conclusion

In this report, we presented four models of statistical analysis of real-time PCR data and one procedure for data quality control. SAS programs were developed for all the applications and a sample set of data was analyzed. The analyses with different models and programs yielded the same estimation of ΔΔCt and similar confidence intervals. The data quality control and analysis procedures will help to establish robust systems to study the relative gene expression with real-time PCR.

## Methods

### Plant material, RNA extraction, real-time PCR and sample data set

The sample data set (Table 1) used for the analysis came from the experiment described below. *Arabidopsis thaliana *(Col1) plants were grown in the growth chamber at 23°C with 14 hours of light for four weeks. Total RNA was isolated with RNeasy Plant Mini Kit (Qiagen, Inc.) from methyl-jasmonate treated *Arabidopsis*, alamethecin treated *Arabidopsis *and control plants, and DNA contamination was removed with an on-column DNase (Qiagen, Inc.) treatment. One microgram of total RNA was synthesized into first strand cDNA in a 20 μL reaction using iScript cDNA synthesis kit (BioRad Laboratories). cDNA was then diluted into 10 ng/μL, 2 ng/μL, 0.4 ng/μL and 0.08 ng/μL concentration series. Three replicates of real-time PCR experiments were performed for each concentration using an ABI 7000 Sequence Detection System from Applied Biosystems (Applied Biosystems). Ubiquitin was used as the reference gene, and the primer sequences for *Arabidopsis *ubiquitin gene were CACACTCCACTTGGTCTTGCG (F) and TGGTCTTTCCGGTGAGAGTCTTCA (R). The primers for target gene (MT_7) were designed by Primer Express software (Applied Biosystems) and the sequences were CCGCGGTACAAACCTTAATT (F) and TGGAACTCGATTCCCTCAAT (R). MT-7 gene is the *Arabidopsis thaliana *gene At3g44860 encoding a protein with high catalytic specificity for farnesoic acid [22]. Primer titration and dissociation experiments were performed so that no primer dimmers or false amplicons will interfere with the result. After the real-time PCR experiment, Ct number was extracted for both reference gene and target gene with auto baseline and manual threshold.

### Real-time PCR experimental design, data output, transformation, and programming

A main limitation of efficiency calibrated method and ΔΔCt method is that only one set of cDNA samples are employed to determine the amplification efficiency. It was assumed that the same amplification efficiency could be applied to other cDNA samples as long as the primers and amplification conditions are the same. However, amplification efficiency not only depends on the primer characteristics, but also varies among different cDNA samples. Using a standard curve for only one set of tested samples to derive the amplification efficiency might overlook the error introduced by sample differences. In our experimental design, we have performed standard curve experiments with four concentrations of three replicates for all samples and genes involved. The ΔΔCt will derive from the standard curves only, and the data quality is examined for each gene and sample combination. The analysis of two samples is presented in the paper as an example. A minimal of PCRs of two replicates in three concentrations will be required for each sample. Even though more effort is required, the data is more reliable out of stringent data quality control and data analysis based on statistical models.

The output dataset included Ct number, gene name, sample name, concentration and replicate. We used Microsoft^® ^Excel to open the exported Ct file from an ABI 7000 sequence analysis system and then to transform data into a tab delimited text file for SAS processing. The sample data set is shown in Table 1.

All programs were developed with SAS 9.1 (SAS Institute).

## Authors' contributions

JSY carried out the real-time PCR experiments, developed the statistical model and SAS programs for analysis, and drafted the article. AR provided assistance in SAS programming and data modeling. FC provided assistance in real-time PCR experiments. CNS provided oversight of the work, conceptualized non-parametric elements, and finalized the draft.

## Supplementary Material

Additional file 1 The SAS program implements the data quality control processes. Click here for file

Additional file 2 The data provides the input data for Program1_QC.sas and Program3_ANCOVA.sas. The data has grouped the Ct values according to the different combination of sample and gene. Click here for file

Additional file 3 The abbreviated SAS output for all the analyses. Click here for file

Additional file 4 The SAS program implements the multiple regression model and derives ΔΔCt. Click here for file

Additional file 5 The input data for Program2_MR.sas, which has contains sample, gene, concentration and Ct number. Click here for file

Additional file 6 The SAS program implements the ANCOVA model and derives ΔΔCt. Click here for file

Additional file 7 The SAS program performs both student t test and Wilcoxon two group tests on the ΔCt to derive ΔΔCt. Click here for file

Additional file 8 The SAS program is a macro that derives confidence interval for Wilcoxon two group tests. Click here for file

Additional file 9 The input data for Program4_TW.sas. The data contains only sample name and ΔCt. Click here for file

Additional file 10 The SAS program performs test for equal slope, grouped slope and adjusted ΔΔCt. Click here for file

Additional file 11 The input data for Program5_LowQualityData.sas. Click here for file
