# Supplementary material for: Statistical analysis of real-time PCR data
Source: BMC Bioinformatics. 2006 Feb 22;7:85. doi: 10.1186/1471-2105-7-85 (PMC1395339; doi:10.1186/1471-2105-7-85)
Supplement: Additional file 3 — The abbreviated SAS output for all the analyses. [file 1471-2105-7-85-S3.doc]

**Abbreviated SAS output for Data Quality Control:**

Real-time PCR data Analysis

Correlation model for slope estimation

------------------------------------------- Class=1 -------------------------------------------

The Mixed Procedure

Solution for Fixed Effects

Standard

Effect Estimate Error DF t Value Pr > |t| Alpha Lower Upper

Intercept 26.7618 0.1256 10 213.13 <.0001 0.1 26.5342 26.9894

lcon -1.0454 0.04828 10 -21.65 <.0001 0.1 -1.1329 -0.9579

------------------------------------------- Class=2 -------------------------------------------

The Mixed Procedure

Solution for Fixed Effects

Standard

Effect Estimate Error DF t Value Pr > |t| Alpha Lower Upper

Intercept 24.8713 0.04237 10 586.97 <.0001 0.1 24.7945 24.9481

lcon -0.9707 0.01629 10 -59.59 <.0001 0.1 -1.0003 -0.9412

------------------------------------------- Class=3 -------------------------------------------

The Mixed Procedure

Solution for Fixed Effects

Standard

Effect Estimate Error DF t Value Pr > |t| Alpha Lower Upper

Intercept 23.1239 0.07664 10 301.70 <.0001 0.1 22.9850 23.2628

lcon -1.0297 0.02947 10 -34.94 <.0001 0.1 -1.0831 -0.9763

------------------------------------------- Class=4 -------------------------------------------

The Mixed Procedure

Solution for Fixed Effects

Standard

Effect Estimate Error DF t Value Pr > |t| Alpha Lower Upper

Intercept 21.9114 0.05757 10 380.59 <.0001 0.1 21.8071 22.0158

lcon -0.9974 0.02213 10 -45.06 <.0001 0.1 -1.0375 -0.9573

**Abbreviated SAS output for multiple regression analysis:**

Analyze Real-time PCR data with SAS 10

Approach I Multiple Regression

Estimation of Treatment and GeneName Effects

The GLM Procedure

Dependent Variable: Ct

Standard

Parameter Estimate Error t Value Pr > |t|

Treatment and Gene Effect -0.68482500 0.11848726 -5.78 <.0001

**Abbreviated SAS output for ANCOVA analysis:**

ANCOVA

Estimate intercept difference

The Mixed Procedure

Estimates

Standard

Label Estimate Error DF t Value Pr > |t| Alpha Lower Upper

Intercepts -0.6848 0.1185 32 -5.78 <.0001 0.05 -0.9262 -0.4435

**Abbreviated SAS output for t-test:**

Analyze Real-time PCR data 1

T-Test

The TTEST Procedure

Statistics

Lower CL Upper CL Lower CL Upper CL

Variable Treatment N Mean Mean Mean Std Dev Std Dev Std Dev Std Err

deltaCt Control 12 3.4136 3.6404 3.8673 0.2529 0.357 0.6062 0.1031

deltaCt Treatmen 12 2.7803 2.9556 3.1309 0.1954 0.2759 0.4684 0.0796

deltaCt Diff (1-2) 0.4147 0.6848 0.955 0.2468 0.3191 0.4516 0.1303

**Abbreviated SAS output for Wilcoxon test:**

Wilcoxon Two-Sample Test

Statistic (S) 220.0000

Normal Approximation

Z 4.0126

One-Sided Pr > Z <.0001

Two-Sided Pr > |Z| <.0001

t Approximation

One-Sided Pr > Z 0.0003

Two-Sided Pr > |Z| 0.0005

Exact Test

One-Sided Pr >= S 1.479E-06

Two-Sided Pr >= |S - Mean| 2.958E-06

Distribution-Free Confidence Interval For Delta Alpha=.95 3

Ref: Hollander and Wolfe, 1973, page 78

Obs lower median upper

1 0.4227 0.6354 0.8805

**Abbreviated SAS output for data quality control for LowQualityData:**

------------------------------------ Class=2 --------------------------------------------

The Mixed Procedure

Solution for Fixed Effects

Standard

Effect Estimate Error DF t Value Pr > |t| Alpha Lower Upper

Intercept 22.4296 0.1177 4 190.50 <.0001 0.05 22.1027 2.7565

lcon -1.1430 0.04560 4 -25.07 <.0001 0.05 -1.2696 -1.0164

**Abbreviated SAS output for type III sums of squares:**

For LowQualityData whole dataset:

Source DF Type III SS Mean Square F Value Pr > F

lcon 1 70.22062905 70.22062905 3408.92 <.0001

Class 3 40.33311310 13.44437103 652.67 <.0001

lcon*Class 3 0.22369356 0.07456452 3.62 0.0362

For Gene1 (control gene):

Source DF Type III SS Mean Square F Value Pr > F

lcon 1 37.41384505 37.41384505 1313.14 <.0001

Class 1 2.54951731 2.54951731 89.48 <.0001

lcon*Class 1 0.12172178 0.12172178 4.27 0.0726

For Gene2 (target gene):

Source DF Type III SS Mean Square F Value Pr > F

lcon 1 32.87996778 32.87996778 2587.69 <.0001

Class 1 2.02308898 2.02308898 159.22 <.0001

lcon*Class 1 0.02878800 0.02878800 2.27 0.1707

**Abbreviated SAS output for the amplification efficiency calculation of LowQualityData dataset:**

Standard

Effect Estimate Error DF t Value Pr > |t| Alpha Lower Upper

Intercept 21.7008 0.3145 10 69.00 <.0001 0.05 21.0000 22.4016

lcon -1.0813 0.1218 10 -8.88 <.0001 0.05 -1.3527 -0.8099

Standard

Effect Estimate Error DF t Value Pr > |t| Alpha Lower Upper

Intercept 25.5609 0.2992 10 85.43 <.0001 0.05 24.8943 6.2275

lcon -1.0137 0.1159 10 -8.75 <.0001 0.05 -1.2718 -0.7555

**Abbreviated SAS output for ΔΔCt estimation of LowQualityData dataset:**

Estimates

Standard

Label Estimate Error DF t Value Pr > |t| Alpha Lower Upper

Intercepts -1.0901 0.05897 6 -18.49 <.0001 0.05 -1.2344 -0.9458

Contrasts

Num Den

Label DF DF F Value Pr > F

Intercepts 1 6 341.71 <.0001
